# Mapping crown rust resistance in the oat diploid accession PI 258731 (*Avena strigosa)*

**DOI:** 10.1371/journal.pone.0295006

**Published:** 2024-02-02

**Authors:** Rawnaq N. Chowdhury, Tyler Gordon, Md. Ali Babar, Stephen A. Harrison, Shahryar F. Kianian, Kathy Esvelt Klos

**Affiliations:** 1 Oak Ridge Institute for Science and Education (ORISE) Research Participant, Small Grains and Potato Germplasm Research Unit, Aberdeen, Idaho, United States of America; 2 USDA-ARS, Plant Genetic Resources Unit, Geneva, New York, United States of America; 3 Department of Agronomy, UFL, Gainesville, Florida, United States of America; 4 School of Plant, Environmental and Soil Sciences, LSU, Baton Rouge, Louisiana, United States of America; 5 USDA-ARS Cereal Disease Laboratory, St. Paul, MN, United States of America; 6 USDA-ARS, Small Grains and Potato Germplasm Research Unit, Aberdeen, Idaho, United States of America; National Bureau of Plant Genetic Resources, INDIA

## Abstract

Oat crown rust, caused by *Puccinia coronata* Corda f. sp. *avenae* Eriks. (*Pca*), is a major biotic impediment to global oat production. Crown rust resistance has been described in oat diploid species *A*. *strigosa* accession PI 258731 and resistance from this accession has been successfully introgressed into hexaploid *A*. *sativa* germplasm. The current study focuses on 1) mapping the location of QTL containing resistance and evaluating the number of quantitative trait loci (QTL) conditioning resistance in PI 258731; 2) understanding the relationship between the original genomic location in *A*. *strigosa* and the location of the introgression in the *A*. *sativa* genome; 3) identifying molecular markers tightly linked with PI 258731 resistance loci that could be used for marker assisted selection and detection of this resistance in diverse *A*. *strigosa* accessions. To achieve this, *A*. *strigosa* accessions, PI 258731 and PI 573582 were crossed to produce 168 F_5:6_ recombinant inbred lines (RILs) through single seed descent. Parents and RILs were genotyped with the 6K Illumina SNP array which generated 168 segregating SNPs. Seedling reactions to two isolates of *Pca* (races TTTG, QTRG) were conditioned by two genes (0.6 cM apart) in this population. Linkage mapping placed these two resistant loci to 7.7 (QTRG) to 8 (TTTG) cM region on LG7. Field reaction data was used for QTL analysis and the results of interval mapping (MIM) revealed a major QTL (QPc.FD-AS-AA4) for field resistance. SNP marker assays were developed and tested in 125 diverse *A*. *strigosa* accessions that were rated for crown rust resistance in Baton Rouge, LA and Gainesville, FL and as seedlings against races TTTG and QTRG. Our data proposed SNP marker GMI_ES17_c6425_188 as a candidate for use in marker-assisted selection, in addition to the marker GMI_ES02_c37788_255 suggested by Rine’s group, which provides an additional tool in facilitating the utilization of this gene in oat breeding programs.

## Introduction

Crown rust, caused by the fungal pathogen *Puccinia coronata* Corda f. sp. *avenae* Eriks. (*Pca*), is a serious threat to oats grown in North America [1–4]. The disease also affects oat production in most oat producing countries [5, 6]. Crown rust affects the photosynthetic ability of the plants by the formation of uredinia on leaves and leaf sheaths, thereby reducing grain filling and lowering grain yield [7, 8]. Crown rust resistance can be qualitative (i.e. a major race-specific gene with resistance based on a gene for gene interaction) [9] or quantitative (i.e., controlled by numerous minor genes largely without race specificity, each contributing only partial resistance) [10, 11]. Race-specific resistance can be characterized by the development of a hypersensitive reaction in an incompatible host-pathogen interaction and is functional at the seedling stage and throughout plant development [8].

Resistance genes come from different oat species and have been documented in the wild hexaploid oat *A*. *sterilis* L., [12], the tetraploid *A*. *magna* Murphy and Terrell [13], and the diploid *A*. *strigosa* Schreb. [14, 15]. More than 45 effective *Pc* genes were obtained from *A*. *sterilis*, including *Pc*38, *Pc*39, *Pc*68, and 19 loci have been identified as derived from the diploid *A*. *strigosa*. Several other wild oat species have been screened and used to identify new resistance genes [16–20]. For example, in a test of 1,099 *A*. *barbata* accessions [21] from the Mediterranean region, 18 accessions were identified as sources of resistance at both seedling and adult stages. In another study [22], Thirty-six out of six hundred seven *A*. *sativa* accessions from diverse geographic locations were identified as a source of resistance for both seedling and adult stages. Thus, *Avena* related wild species have been and continue to be an important source of resistance which will be demonstrated by talking about a novel highly effective crown rust resistance from PI 258731 accession.

Rines et al. [23] introgressed crown rust resistance from PI 258731 using a bulk segregation analysis (BSA) that assumed a single gene was derived from PI 258731. In addition to the major QTL identified by BSA there was a potential for other QTL undetectable by this method. Such limitation of the BSA strategy might directly target the resistance QTL with major effects, not those with minor effects. For example, initial characterization of Barley Stem Rust resistance (BSR) in the barley line Grannelose Zwiezelige (GZ) was performed using BSA [24]. The major QTL identified by that work was later found to be accompanied by 3 minor QTL after linkage mapping using RILs. Our study performed linkage mapping in resistant by susceptible *A*. *strigosa* cross utilizing a marker set from *A*. *sativa* to verify the number and effect size of QTL locus in PI 258731 accession.

In this study our objectives were 1) to map the location of a QTL conferring crown rust resistance in the PI 258731 line; 2) to study the relationship between original genomic location in *A*. *strigosa* and the location of the introgression in *A*. *sativa* genome; and 3) to identify molecular markers highly linked to the PI 258731 resistance QTL that could be used to refine marker assisted selection.

## Materials and methods

### Plant material

PI 573582 is an *A*. *strigosa* accession originally received by the U.S. National Plant Germplasm System (NPGS) in 1992 from the N.I. Vavilov Institute of Plant Industry, and a single plant selection from this accession was used as the susceptible female parent in this study. A single plant selection of PI 258731, obtained from the USDA-ARS National Small Grains Collection maintained at Aberdeen, Idaho (http://www.ars.grin.gov), was used as the pollen parent and source of crown rust resistance. This *A*. *strigosa* accession was originally received by the National Plant Germplasm System (NPGS) in 1959 from Galicia, Spain. In our study, PI 573582was crossed with PI 258731and the progenies were advanced through single seed descent to produce 168 F_5:6_ recombinant inbred lines (RILs) with the resulting population denoted as 573582/*258731*.

To identify the phenotypic reactions (seedling and field reaction) of our two parental lines, we screened the parental lines along with 125 *Avena strigosa* accessions available from the NSGC by using two *Pca* races (TTTG and QTRG). The 125 *Avena strigosa* accessions were also tested in the field to assess the correspondence between marker genotype and phenotype.

### Phenotyping

Seedling tests were carried out using the *P*. *coronata* f. sp. *avenae* races TTTG and QTRG (designated using the race nomenclature of [25]) at the Small Grains and Potato Germplasm Research Facility of the USDA in Aberdeen, Idaho in 2021–2022. Seedlings of each parent and RILs were grown separately for each *Pca* races tested. A single pustule isolate from each race was propagated on a susceptible oat line (Ajay, 74AB1952/74AB2608, F_5_ Selection) and the inoculated plants were used to collect inoculum for seedling resistance testing. Both *Pca* races of crown rust were virulent against PI 573582, but avirulent against PI 258731 and were used to inoculate seedlings of the 573582/*258731* population in two replications for each. Within each test, two to three seeds per RIL were planted into cone-tainers (3.8 mm diameter × 210 mm depth, Stuewe & Sons, Inc., Tangent, OR) filled with a mix of sand, peat moss, and vermiculite mixed by volume at 3:2:2 and maintained in a greenhouse adjusted to 22°C and a 16-hour photoperiod. Three replicates of each parent were included in each test, and the planting order was randomized within each replication. Two weeks after planting, seedlings were inoculated at a concentration of 1 × 10^5^ spores ml^-1^ inoculum suspension [26] of the respective *Pca* race suspended in Soltrol 170 isoparaffin (Chevron Phillips, The Woodlands, TX) using a motorized sprayer (GAST Manufacturing inc., Benton Harbor, MI) and a small atomizer (G-R Manufacturing Manhattan, KS). Plants were left in the open air for an hour to dry, and then transferred to a dew chamber set at 20°C with no light. After 18 hours in the dew chamber, plants were placed in a growth chamber adjusted to 20–22°C with an 18-hour photoperiod. Disease reactions on the first (leaf emerged from the 1^st^ node) and second (leaf emerged from the 2^nd^ node) leaves [27] were recorded 14 days after inoculation as Infection Type (IT) on a 0 to 4 scale where 0 (immune),;(fleck), 1, and 2 are considered resistant reactions, while 3–4 are susceptible reactions [22, 25, 28].

Crown rust infection reactions were assessed in field trials in 2021 at the University of Minnesota Matt Moore buckthorn nursery, Saint Paul, MN; and in 2022 at the Louisiana State University Central Research Station, Baton Rouge, LA and at the University of Florida crown rust field nursery, Gainesville, FL. The field experiments in 2021–22 were conducted in a complete randomized block design with two replicates. Parents were randomized within each field replicates. Disease reaction observations were made at the milk to dough stages on a per head row basis using the modified Cobb scale [29]. Reactions to crown rust were evaluated by assigning each plot at Louisiana State University (LSU) and University of Florida (UFL) with a disease severity (estimation of the percentage of leaf area covered by pustules) rated visually on a 0 to 100 scale, and an infection reaction (IR) rated as resistant = 0, resistant to moderately resistant = 0.2, moderately resistant = 0.4; moderately resistant to moderately susceptible = 0.6, moderately susceptible = 0.8, moderately susceptible to susceptible = 0.9, and susceptible = 1.0. Disease severity (DS) in the MN nursery (2021) was rated visually as 0 = 1, low = 2, medium = 3, medium to high = 4 and high = 5. DS was then multiplied by IR to obtain the coefficient of infection (CI) [30]. The CI, DS, and IR data were used for QTL mapping.

### Genotyping

Fourty-six RILs that were susceptible to *Pca* race TTTG and QTRG (ITs 3 to 3+) and 48 RILs that were resistant (ITs of 0; to 2N) to *Pca* race TTTG and QTRG across the four replications were chosen for genotyping. After disease symptoms were rated, leaf tissue (2 cm) was collected from the third or fourth leaves (i.e., non-infected), then freeze dried for 5 to 7 days at -50°C (Labconco). Freeze dried samples from the RILs and the parents were sent to the USDA-ARS Small Grains Genotyping Laboratory in Fargo, ND and genotyped using the 6K oat Infinium iSelect SNP assay as described by the manufacturer (Illumina, San Diego, CA). Genotype calling was performed automatically using the DBSCAN procedure in Genome Studio, v. 2.0 (Illumina, San Diego, CA, 2016). Within the 573582/*258731* (diploid *A*. *strigosa*) population, 4645 of the 4852 Illumina SNP markers either did not amplify or were monomorphic (markers with allele frequencies of either 1 or 0). Markers were also removed from analysis if missing calls were ≥10% and heterozygosity was ≥21%. A final set of 168 SNPs were obtained on 94 RILs and the parents.

### Statistical analysis

The goodness-of-fit of the observed disease reaction to the expected segregation ratio of 1:1 and 1:06:1 corresponding a single gene model were tested using Pearson’s chi-squared (χ2) distribution analysis for TTTG and QTRG separately. RILs that differed in seedling reaction to TTTG and QTRG were used to calculate an estimate of the cM distance [total number of recombinant type /(total number of parental types+ total number of recombinant type) *100] between the two resistance genes [31].

Crown rust seedling IT was used to code each RIL as 0 = susceptible, 1 = mixed or segregating, and 2 = resistant. Two linkage maps in response to TTTG and QTRG were developed separately from the genotype data using the software JMP Genomics v.10.0 (SAS Institute, Cary, NC). The initial number of linkage groups and marker order in each linkage groups were determined using the “recombination and linkage groups”, and the “linkage map order” functions of the software, respectively. SNPs were placed into 7 linkage groups using a maximum distance between markers of 39 cM. Genetic distances between SNP markers and resistant loci were calculated in centiMorgans (cM) using the Kosambi map function [32]. The new phenotypic markers were added to the existing linkage group using two-point linkage analysis with a LOD threshold of 3.0 and a maximum distance of 39 cM (Kosambi).

Multiple interval mapping (MIM) with forward selection as implemented in JMP Genomics v.10.0 was performed for each measure of field resistance. After running a permutation test with 1000 iterations, a minimum logarithm of odds (LOD) score of 3.2 was set to determine statistical significance. LOD scores, additive effects, and percent variation explained by each SNP marker were estimated using MIM.

To evaluate whether the QTL influencing field resistance was the same as that mapped using seedling resistance, predicted residuals from each of the field phenotypic variables were calculated using a generalized linear model (GLM) model, Yi = G+E, where Yi = field disease phenotype, G = genotype at the marker nearest to the peak LOD score, E = Error. Residuals were added to the population grand mean to create field phenotypes adjusted for seedling resistance QTL genotype. The new adjusted field disease variables were then used in QTL analysis as above.

### *A*. *strigosa* accessions

Oat3K Infinium genotyping data (USDA_SoywheOatBar-3K) were used for the marker analysis and only markers in the subset selected for PCR allelic competitive extension (PACE) primer design were filtered out. In addition, a total of five PACE markers were designed from the Illumina SNP sequences that were closely linked with either side of the PI 258731 resistance locus using the free assay design service by 3CR Bioscience (Essex, UK) (www.3crbio.com/free-assay-design) to assess the correspondence between marker and trait in a diverse germplasm pool (S4 Table in S1 File). The sequences of the PACE PCR primers are reported in S4 Table in S1 File. The forward and reverse primers were maintained at stock concentrations of 100 μM. In a 100 μl assay mix, 12 μl each of the forward primers was mixed with 30 μl of the reverse primer and 46 μl of DEPC water. The final 10 μl reaction mix per well was composed of the following: 5 μl 2X PACE Genotyping Master Mix (3CR Bioscience, Essex, UK), 2.86 μl DEPC water, 0.14 assay mix, and 2.0 μl template DNA. Cycling conditions were 94°C for 15 min, 10 cycles of 94°C for 20 s and 65°C for 60 s with an annealing temperature decrement of 0.8°C per cycle, and 30 cycles of 94°C for 20 s and 57°C for 60 s. When cycling had ended, assays were read for FAM and HEX fluorescence with a CFX96 (Bio-Rad, Hercules, CA).

Assays were used to genotype 125 diploid *A*. *strigosa* accessions. Two phenotypic groups were assigned based on the IT score when exposed to two *Pca* races. Accessions showing resistance reactions to both races-named as RR group (phenotypically alike the resistant parent) and susceptible reaction to both *Pca* races-named as SS group. Frequency of the resistant parent (PI 258731) allele and mean phenotypic values of field resistance (IR, DS, CI) were calculated for each phenotype group.

## Results

### Phenotypic analysis

**Fig 1:**
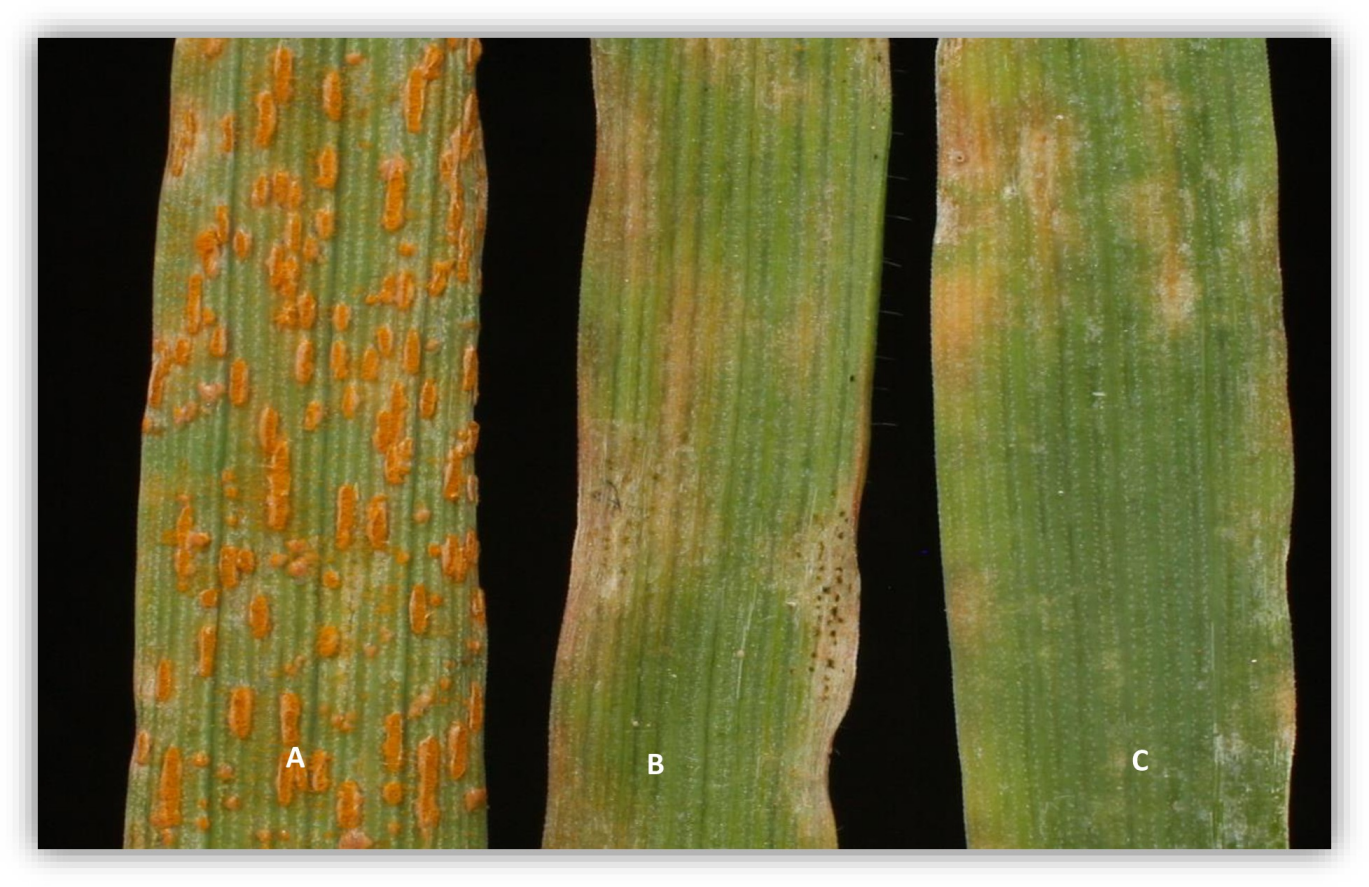
*Avena strigosa* primary leaf infection type (IT) phenotypes inoculated with two *Pca* races TTTG and QTGB and shown 14 dpi; A. Susceptible parent, PI573582, with IT 4 B and C. PI 258731 carrying resistance with two different IT: “; N” (TTTG) and “0N” (QTRG).

Resistant parent (PI 258731) seedlings, when inoculated with *Pca* races TTTG and QTRG, showed ITs of ‘0;N’ to ‘;12N’ and the susceptible parent (PI 573582) had ITs between ‘3’ and ‘3+’ (Fig 1). The 573582/*258731* (RILs) seedling showed ITs of ‘0;N12+’ for resistant reactions, and ITs ‘33+’ for susceptible reactions. We observed 62 resistant lines, 38 segregating lines and 67susceptible lines when inoculated with TTTG and 78 resistant lines, 18 segregating lines and 59 susceptible lines when inoculated with QTRG across four growth chamber trials. We expected to observe 1:06:1 segregation ration in this F_5:6_ population. But there was an excess of segregating lines in this F_5:6_ population which might suggest a greater number of plants escaping infection and/or scoring error and/or the presence of modifier genes (Table 1). Of the remaining resistant (62) and susceptible (67) lines after removal of the segregating lines do fit the expected ratio (1R:1S) for a single gene in response to each of the individual *Pca* races (Table 1). We observed 1 RILs susceptible to TTTG while resistant to QTRG and 2 RIL that was resistant to TTTG while susceptible to QTRG which was evidence of crossing over between the two genes. We estimated that the two genes were 2.9cM apart (S1 Table in S1 File).

**Table 1:** Segregation of F_5:6_ 573582/*258731* recombinant inbred lines (RILs) to two *Pca* races in greenhouse experiments and χ2 test analysis of F_5:6_ progeny.

	Non segregating RILs^b^		χ² values^d^			Segregating RILs^c^	χ² values ^e^		
**Race ^a^**	Resistant	Susceptible	Ratio	χ2	*P* value		Ratio	χ2	*P* value
**TTTG**	62	67	1:1	0.12	0.72 ns	38	1:06:1	83.2	≤0.001
**QTRG**	78	59	1:1	2.63	0.10 ns	18	1:06:1	11.1	0.003

^a^ The greenhouse experiments were conducted in a complete randomized block design with two replicates for each of the two *Pca* races (TTTG and QTRG). Parents were randomized within each greenhouse replicate.

^b^ RILs were judged to be non-segregating based on seedling resistant reaction consistent between the two *Pca* races.

^c^ RILs were judged to be segregating based on seedling resistance reaction differed between the two *Pca* races.

^d^ Chi-square values were not significantly different than the indicated ratios at *P* = 0.05, df = 1.

^e^ Chi-square values were significantly different than the indicated ratios at *P* = 0.05, df = 1.

Approximately 87% of the unrelated *A*. *strigosa* accessions were resistant to *Pca* race TTTG with ITs between ‘0;’ and ‘;2N’ whereas 49% of the accessions were resistant to *Pca* race QTRG with ITs ‘33+’ (S2 Table in S1 File). All summarized seedling and field data from the 125 *A*. *strigosa* accessions has been deposited into the GRIN-Global database: https://npgsweb.ars-grin.gov/gringlobal/search. *A*. *strigosa* accessions with a phenotype like PI 258731 for both *Pca* races were postulated to carry PI 258731 loci (RR group) while those with phenotypes dissimilar for one or both *Pca* races were presumed not to carry the PI 258731 resistance loci (SS group). We identified fifty-eight of *A*. *strigosa* accessions that showed clear resistant ITs of 0; to;2N to *Pca* race TTTG and QTRG, classified as the RR group. Fourteen accessions were susceptible (ITs: 33+) to both *Pca* race TTTG and QTRG and were classified as SS (Table 2). The rest of the accessions that had ambiguous scores were not included in the analysis.

**Table 2:** Phenotypic grouping of *A*. *strigosa* accessions by reaction to *Pca* races (TTTG and QTRG); mean phenotypic values of field resistance (IR, DS, CI) in each phenotypic group.

		*Pca* race		Field resistance		
**Group**	No. of accessions	TTTG_ITs	QTRG_ITs	Mean IR	Mean DS	Mean CI
**SS**	14	33+	33+	0.9	69.1	66
**RR**	58	;0N	;2N	0.6	37.6	29.9

**Fig 2:**
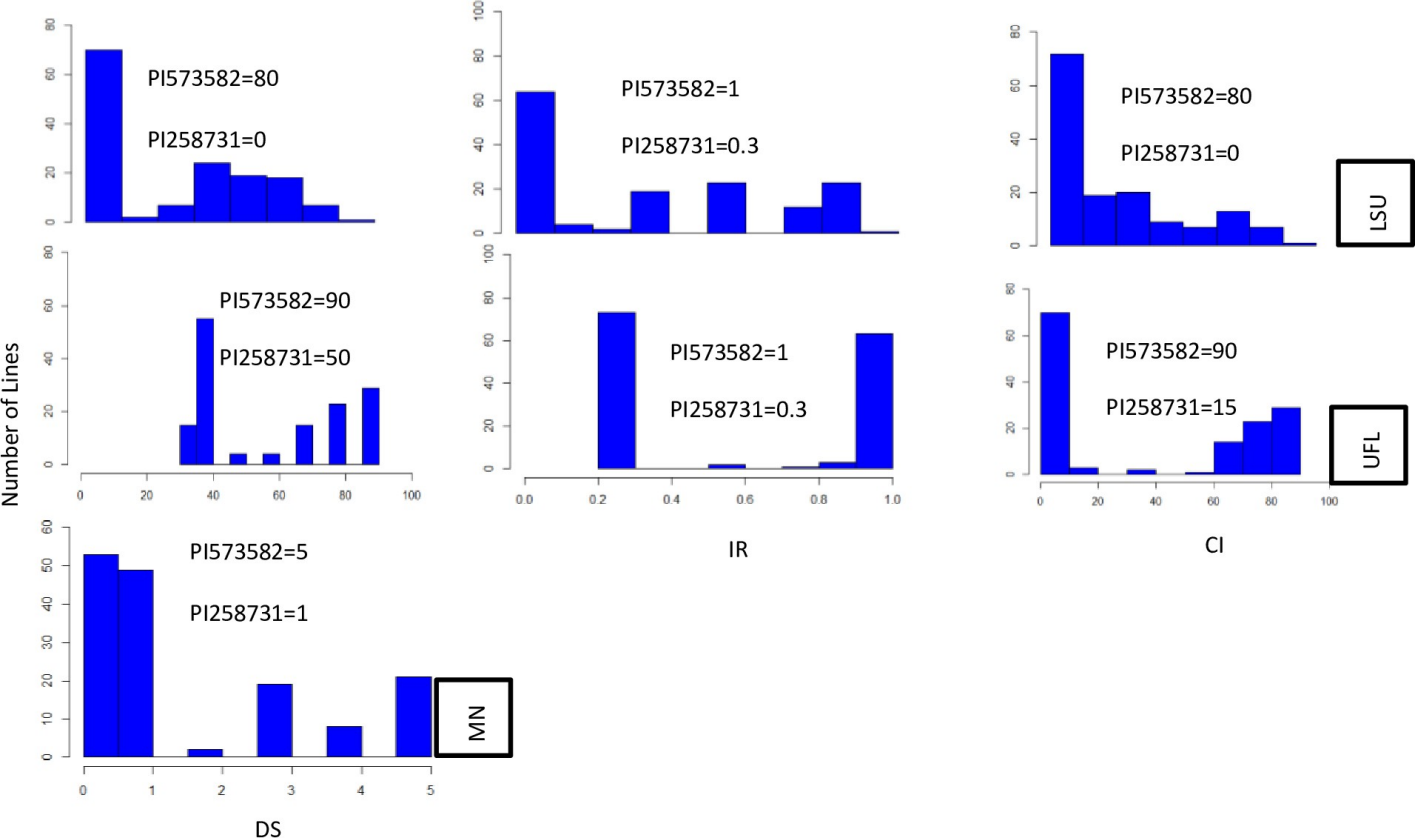
Phenotypic scores for disease severity (DS) at LSU, UFL and MN locations and phenotypic scores for infection reaction (IR) and coefficient of infection (CI) at LSU and UFL locations in the RIL population. The y-axis in each plot represents the number of lines from the population that display scores shown in the x-axis, while locations are shown on the right y-axis; LSU = Louisiana State University Central Research Station in Baton Rouge, LA, UFL = University of Florida crown rust field nursery in Gainesville, FL, MN = University of Minnesota Matt Moore buckthorn nursery in Saint Paul, MN. Mean score of the parents is indicated in the legend.

Phenotypic data on the 573582/*258731* population as well as 125 *A*. *strigosa* accessions were collected in 2021 from the Matt Moore buckthorn nursery in Saint Paul, MN (DS), and in 2022 from LSU and UFL field nurseries (DS, IR, and CI). The 573582/*258731* RILs exhibited a distribution skewed towards resistant phenotypes at the LSU location, whereas bimodal distributions were observed at the UFL location for DS, IR and CI (Fig 2). The phenotypic values for the susceptible parent, PI 573582 ranged from 80–90 for DS, 1.0 for IR, and 80–90 for CI at LSU and UFL locations. The range of phenotypic values for the crown rust resistant parent, PI 258731 were 0–50 for DS, 0.3 for IR, and 0 to 15 for CI at LSU and UFL locations (Fig 2). At MN (2021), the phenotypic value for the crown rust resistant parent (PI 258731) was 1 = low infection and for the susceptible parent (PI 573582) was 5 = high infection showing a distribution skewed towards resistant phenotypes.

The RR group of the *A*. *strigosa* accessions displayed an average field phenotypic score of 0.6, 37.6 and 29.9 for IR, DS and CI, respectively, in the LSU and UFL field nurseries, while the SS group had average field phenotypic scores of 0.9, 69.1 and 66 in the same field nurseries. Resistant and susceptible parents were easily distinguished by all phenotypic measures in all locations (Fig 2 and Table 2).

### Linkage mapping

**Fig 3:**
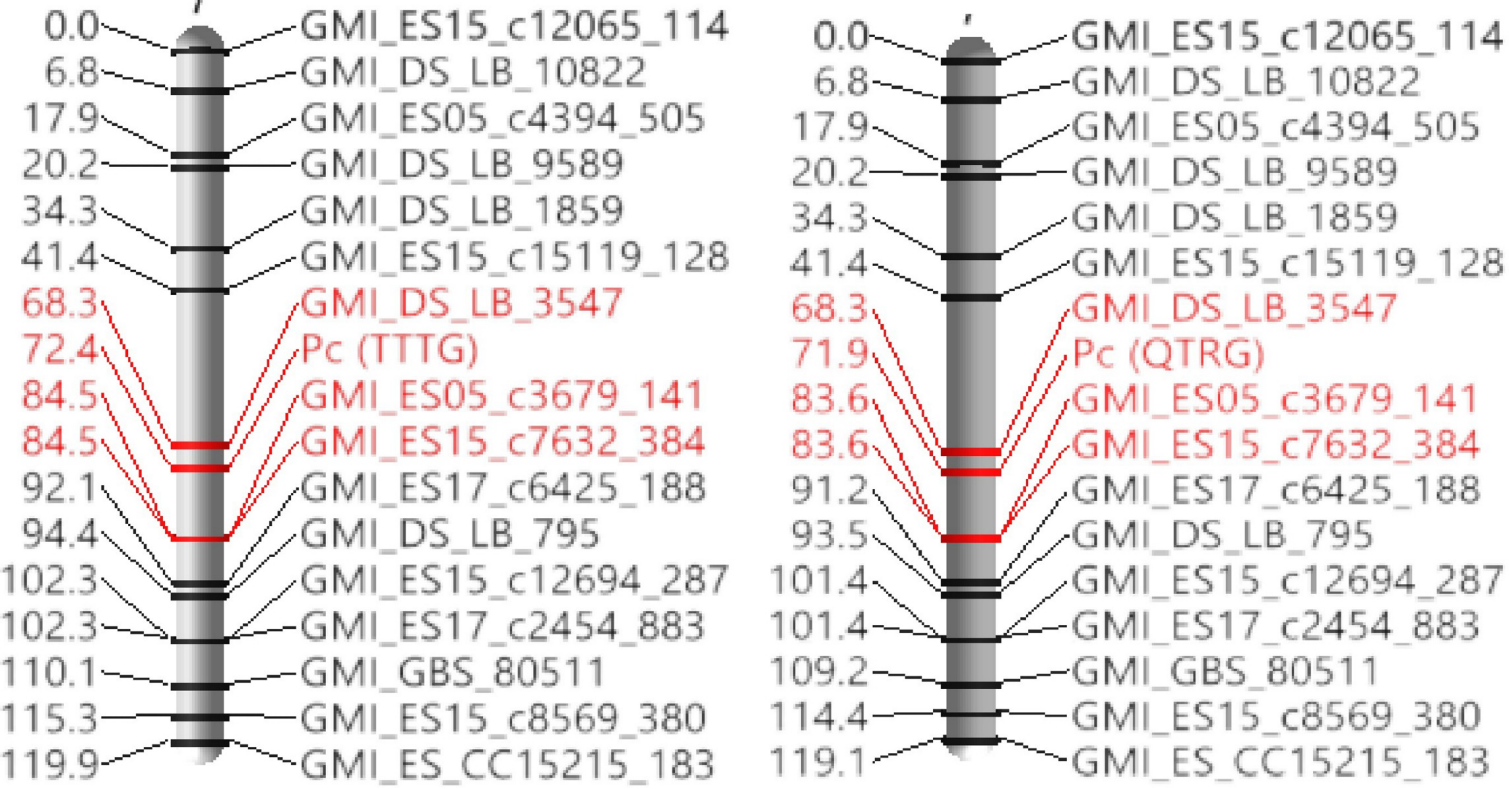
**a**. Genetic linkage map of *Pc* (TTTG) resistance constructed with Illumina 6K SNPs and oat F_5:6_ RILs derived from a cross between PI 258731 and PI 573582. The flanking SNP markers are highlighted in red color. **b**. Genetic linkage map of *Pc* (QTRG) resistance constructed with Illumina 6K SNPs and oat F_5:6_ RILs derived from a cross between PI 258731 and PI 573582. The flanking SNP markers are highlighted in red color.

A sparse genetic map with seven linkage groups corresponding to the seven chromosomes of the haploid genome, were constructed from 168 polymorphic SNPs in the 573582/*258731* F_5:6_ bi-parental population in response to the two *Pca* races separately. Based on the linkage analysis two separate genetic regions (0.6cM apart) on LG7 were identified. The total length of the two maps were 657.8 to 659.2 cM with a mean of 0.3 markers per cM corresponding to TTTG and QTRG, respectively. LG2 had the most SNP markers with 41 markers for both the linkage map (S3 Table in S1 File). The resistance locus was mapped to 72.4 cM, between markers GMI_DS_LB_3547 (at 68.3 cM) and GMI_ES05_c3679_141, GMI_ES15_c7632_384 (at 84.5 cM) on LG 7 and to 71.8 cM, between markers GMI_DS_LB_3547 (at 68.3 cM) and GMI_ES05_c3679_141, GMI_ES15_c7632_384 (at 83.0 cM) on LG 7 in response to the two *Pca* races TTTG and QTRG, respectively (Fig 3A and 3B).

### QTL mapping

**Fig 4:**
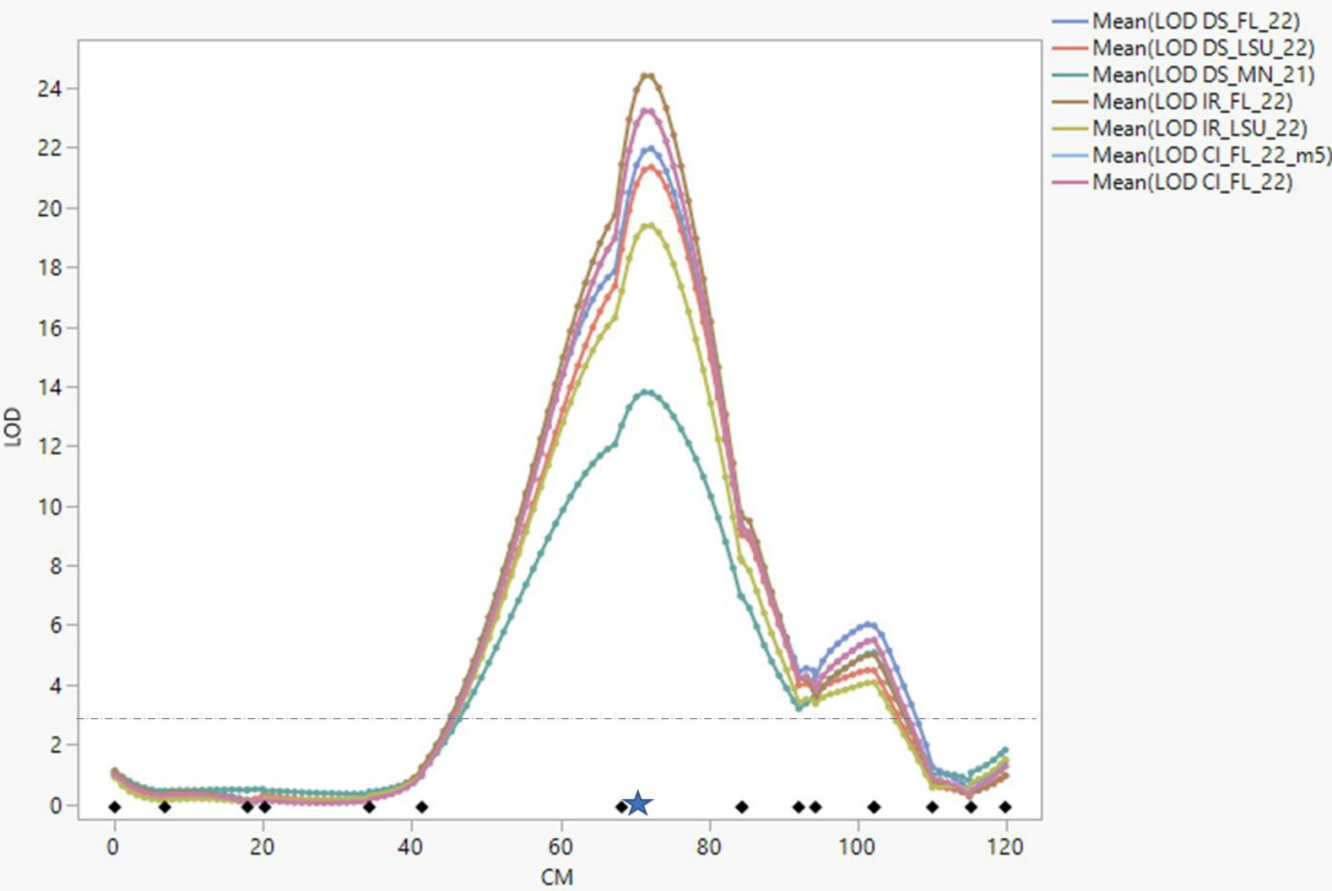
Logarithm of odds (LOD) score profiles of quantitative trait loci (QTL) for field resistance, colored dotted lines on linkage group 07 based on multiple interval mapping. Linkage group 07 corresponds to 573582/*258731* population in Fig 2. The X-axis indicates cM position of the SNPs. The position of the phenotypic markers is indicated with blue star. A threshold of LOD = 3.2 was determined by a permutation test with 1000 iterations (p<0.05).

**Fig 5:**
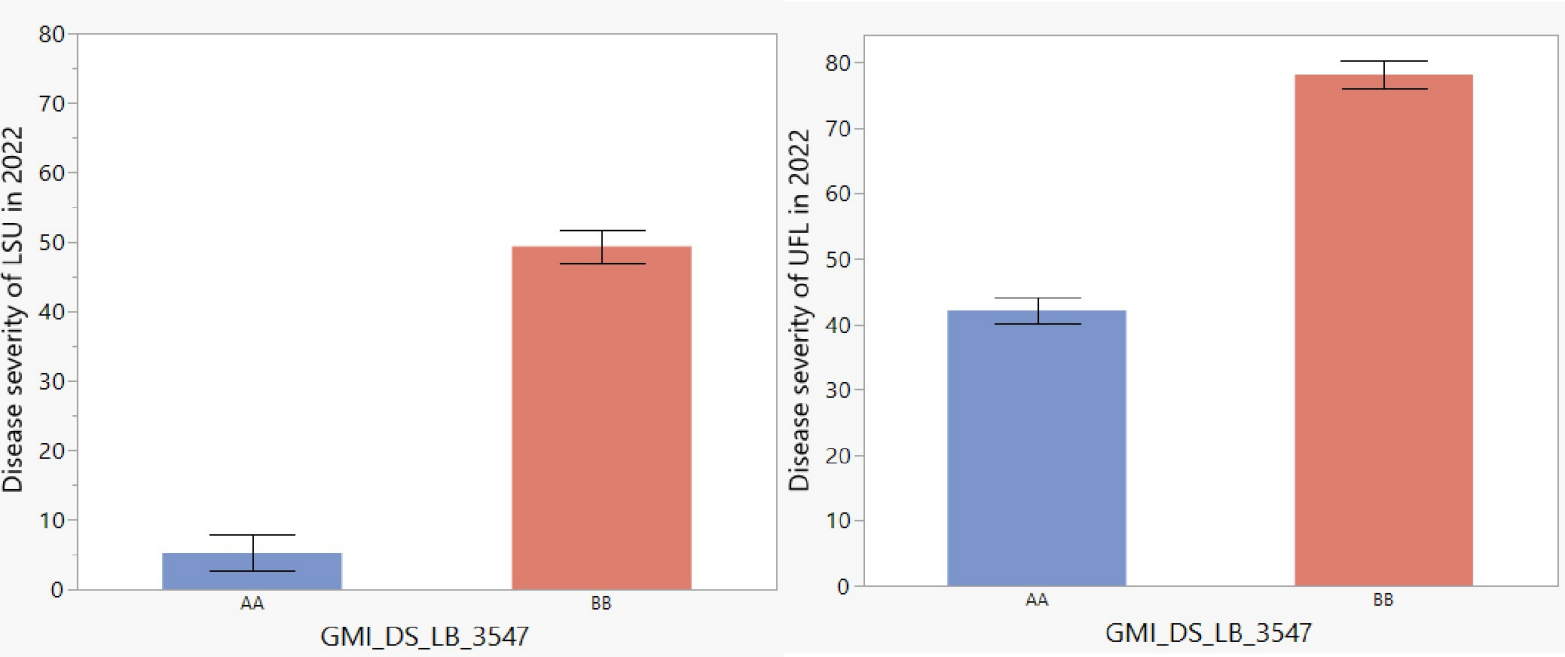
Disease severity effect plots for major QTL (QPc.FD-AS-AA4), based on the allelic distributions determined at the peak SNP marker of the QTL (GMI_DS_LB_3547) at A. LSU location B. UFL location. The two possible alleles are indicated in each group (AA = alleles associated with resistant parent, BB = alleles associated with susceptible parent).

**Fig 6:**
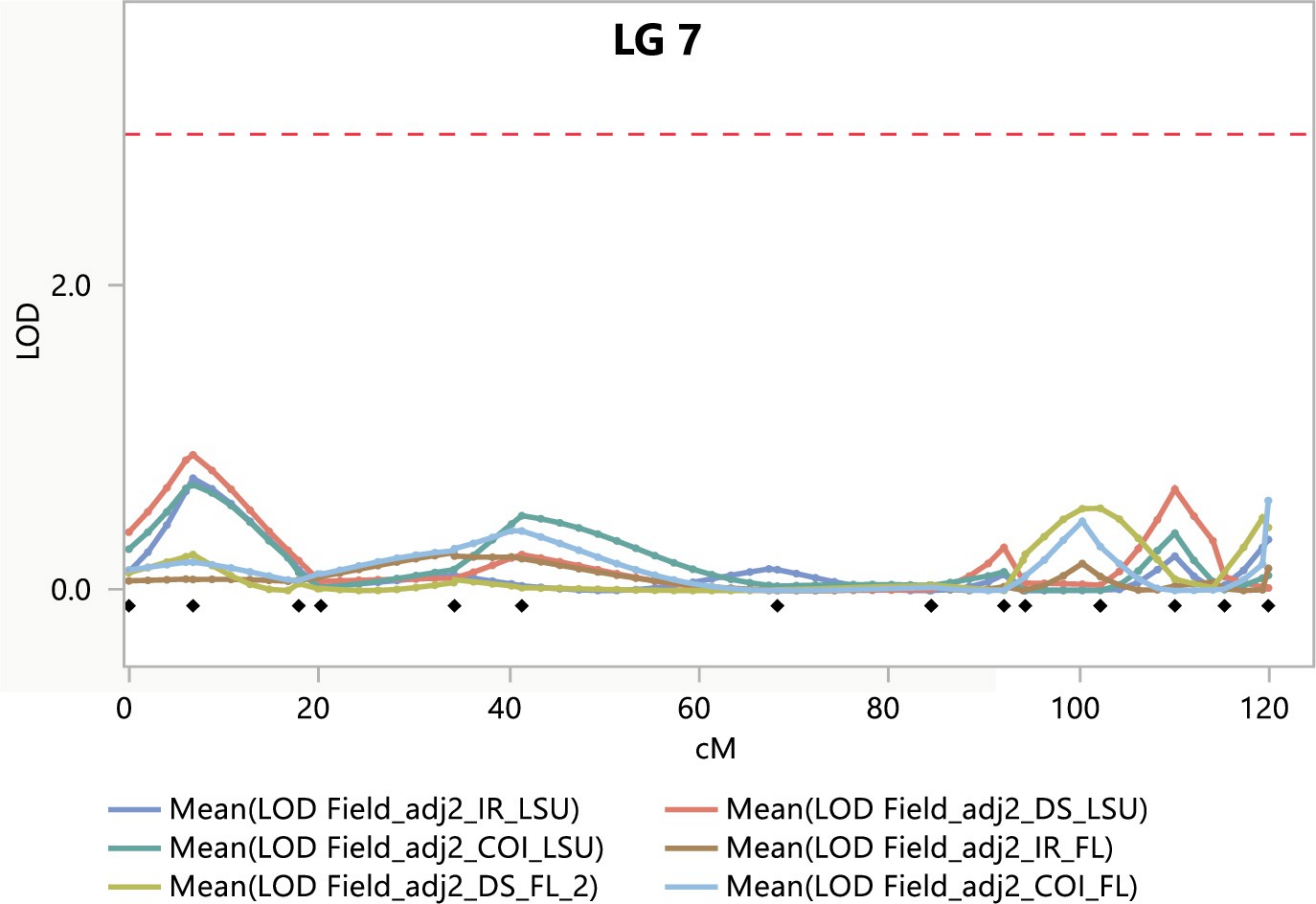
Logarithm of odds (LOD) score profiles of quantitative trait loci (QTL) for field phenotype adjusted for PI 258731 seedling resistance allele, colored dotted lines on linkage group 07 based on multiple interval mapping. A threshold of LOD = 3.2 was determined by a permutation test with 1000 iterations (P<0.05).

MIM identified a major QTL (QPc.FD-AS-AA4) influencing CI, DS, and IR in all environments (Fig 4). We report in detail the results of analyses of DS in the manuscript. The strongest evidence on linkage with QPc.FD-AS-AA4 was LOD = 22.05, 22.06 and 14.79 (in Baton Rouge, Gainesville, and St. Paul, respectively) at 72.4 cM on LG7. QPc.FD-AS-AA4 explained an average of 61.22% of the DS variation over the three environments. Negative QTL effects for DS indicated that resistance alleles were contributed by PI 258731, the resistant parent, which was consistent with the absence of clear transgressive segregants for DS in the RILs (Fig 2). Genotype classes of the closely linked SNP (GMI_DS_LB_3547) for QTL QPc.FD-AS-AA4 showed significant differences (P = <0.001) in DS among the lines containing the resistant parent alleles compared to those that possessed the susceptible alleles (Fig 5). Adjusting DS for PI 258731 seedling resistance allele carrier status removed all evidence of QTL influencing field resistance (Fig 6) suggesting that the seedling resistance genes identified in growth chamber condition were also influencing the crown rust resistance observed in the field evaluations.

#### *A*. *strigosa* accessions

The Illumina 3K (USDA_SoywheOatBar-3K) chip was used to determine the frequency of the resistant allele in oat cultivars (353 spring sown elite oat germplasm of the Collaborative Oat Research Enterprise) (J. Fiedler, personal communications). We filtered out only markers that were used to develop PACE assays (S4 Table in S1 File). Only two markers (GMI_ES15_R7632_384 and GMI_ES17_R6425_188) from our marker subset and marker (GMI_ES02_C37788_255) which was suggested for marker assisted selection by Rines et al. [23] had available genotyping data for the CORE collection (a subset mainly from spring sown oat). The resistant SNPs linked to PI258731 was of relatively low frequency in the subset of spring sown CORE collection with 10% and 13% of cultivars containing the resistant allele for the markers (GMI_ES17_R6425_188 and GMI_ES02_C37788_255), respectively. However, the frequency of resistant SNP was of relatively high in the same CORE sub-population, with 79% of the cultivars containing the resistant SNP for the marker (GMI_ES15_R7632_384). Although, the marker GMI_ES15_R7632_384 (12.1cM) was more closely linked to the two resistance locus compared to the marker GMI_ES17_R6425_188 (19.7 cM), the former marker may not be suitable in all breeding programs.

Five SNPs linked with the resistant locus were used for PACE marker development to predict the performance of these markers in wider *A*. *strigosa* germplasm. All five PACE markers were polymorphic between the resistant locus carrier, PI 258731, and the susceptible *A*. *strigosa* parent PI 573582, but only three of the PACE markers (PACE_GMI_ES15_R7632_384; PACE_GMI_ES05_R3679_141; and PACE_GMI_ES17_R6425_188) were polymorphic among the set of diverse diploid *A*. *strigosa* accessions. Frequency of the resistant parent allele in each of the two phenotypic groups for three putative PACE markers are presented in Table 3. In the RR group, 97% of accessions possessed the same allele as PI 258731 resistant parent allele for the marker PACE_GMI_ES15_c7632_384, but this allele was also present in 86% of SS group accessions. Similar patterns were observed for the other two markers (PACE_ GMI_ES05_c3679_141; PACE_GMI_ES17_c6425_188), suggesting that the markers were not able to define a unique SNP haplotype with our resistant allele in the diverse population.

**Table 3:** Frequency of the resistant parent allele for three putative PACE markers in each phenotypic group.

		Frequency of resistant parent allele		
**Group**	No of accessions	PACE_GMI_ES15_c7632_384	PACE_GMI_ES05_c3679_141	PACE_GMI_ES17_c6425_188
**SS**	14	0.86	0.79	0.43
**RR**	58	0.97	0.83	0.48

## Discussion

In this study we identified the genomic location of a crown rust resistance locus which was introgressed from PI 258731 into *A*. *sativa* by Rines et al. [23]. In addition, we observed differences of phenotype among studies; relationship between the original genomic location in *A*. *strigosa* and the location of the introgression in the *A*. *sativa* genome; markers with potential for use in marker assisted selection; and an inability to ascribe PI258731 resistance as the source of resistance in other *A*. *strigosa* accessions.

In our study, we observed different responses of the resistant parent to the two *Pca* races. We analyzed the seedling score data of the *Pca* races separately and data were found not to fit an expected 1:06:1 segregation ratio for a single gene in in F_5_ derived RILs. We observed that 22% of the RILs were segregating when inoculated with TTTG and 10% when inoculated with QTRG. The higher than expected segregation percentage in the F_5:6_ population suggested that other factors were contributing to the resistance phenotype, but we were unable to dissect the segregation ratio with the number of seedlings available. Excess heterozygosity could be due to plants escaping infection and/or scoring error. However, a Chi-square analysis with of non-segregating RILs only, did fit the expected 1R:1S segregation ratio. Therefore, we performed linkage analysis based on the assumption of a single segregating resistance allele but analyzed separately within the two *Pca* races.

We observed evidence for crossing over when comparing the responses to TTTG and QTRG suggesting two genes for resistance segregating in our population. Unlike Rine’s group [23] where they used field evaluation data of an F2 population (from a resistant by resistant cross) and inferred a single gene was derived from PI 258731 based on the segregation ratio analyzed with a chi-squared test. It is possible that the tightly linked genes were likely appeared as single gene in Rine’s study [23] because they used a single score data for the mixed field races for the analysis. Whereas we used two separate *Pca* races for the evaluation of seeding resistance and infer that two genes were influencing seedling resistance in response to TTTG and QTRG. We were able to get an estimate of the cM distance between the two genes and found that the two genes were 2.9cM apart.

We selected a subset of RILs with clear phenotypes to generate marker data and used them for linkage analysis. Our linkage map with the selected RILs did map, as expected, a single gene for each of the *Pca* races and they were 0.6cM apart. This is consistent with our conclusion from the segregation analysis of the larger population. However, the distance between the two genes was probably more accurately represented in the data used for segregation analysis than the data used for linkage analysis because the estimate based on the non-genotyped data did not impose any selection for unambiguous phenotype. This process may have reduced in the unconscious selection against the crossover recombinants. In addition, power to detect other potential genetic contributors to resistance was also reduced.

In this study, a higher level of resistance was observed in the resistant parent and progeny than reported by Rines et al. [23]. The adult plant resistance of PI 258731 was first noted in the Matt Moore buckthorn nursery in a routine field screening of the USDA oat germplasm collection and in a seedling screening for crown rust resistance [18]. The Cabral et al. [18] observed a moderately susceptible to moderately resistant reactions in PI 258731 at 14 days post inoculation while screening seedling crown rust resistance. In our seedling test, the resistant RILs were moderately resistant to resistant. The differences in the crown rust expression in these studies could be due to differences in assessment method, including rater bias [26], environmental conditions in the respective growth chambers or differences in the virulence composition of the two *Pca* races used [33].

Regardless, screening of PI 258731 in the Matt Moore buckthorn nursery suggests continuing effectiveness of this source of resistance. Additionally, this source of resistance continues to be effective in Australian crown rust nurseries (Robert Park, personal communication). In this study, seedling resistance observed in the growth chamber translated to field resistance at Baton Rouge, LA, Gainesville, FL and the Matt Moore buckthorn nursery, MN. This is consistent with the complete field resistance observed by Rines et al. [23] in hexaploid lines carrying the resistance introgressed from PI 258731. Our observations occurred four years after Rines et al. [23] in the same buckthorn nursery where a sexually recombining crown rust population is present which provides evidence for extending the span of effective years from 8 to 12 years since the first known introduction of this gene to the nursery. Full-genome sequences exist for many of the *A*. *strigosa* accessions in the NSGC. A comparative sequencing approach like that used by Gordon et al. [34] to fine map crown rust in an *A*. *strigosa* accession could be used in conjunction with the phenotypes generated in this study to identify a candidate gene conferring unique and durable resistance to crown rust.

Sixteen SNP markers from the *A*. *sativa* 6K illumina SNP chip were segregating on LG7 between the two *A*. *strigosa* parents. Using the same 6K SNP chip Rines et al. [23] observed 41–50 markers segregating on Mrg20 (corresponding to *A*. *sativa* chromosome 4A) [35]. This included one SNP marker (GMI_ES02_C37788_255) within the inferred region of introgression. The marker inferred by Rines et al. [23] within the region of introgression was amplified in our *A*. *strigosa* parents but was not segregating in the mapping population. However, these maps shared one SNP marker (GMI_ES15_c7632_384) near the two QTL. GMI_ES15_c7632_384 was 12.1 cM from the resistance gene in the *A*. *strigosa* map and 1.6 cM from the introgressed crown rust resistance locus in the *A*. *sativa* map [23]. This is consistent with a chromosome 4A (*A*. *sativa*) introgression from the *A*. *strigosa* LG7 in this study.

Markers linked to the QTL region could be of interest to oat breeders for use in marker assisted selection of this *A*. *strigosa* introgression within *A*. *sativa* cultivar development. Marker (GMI_ES02_C37788_255) was suggested by Rines et al. [23] based on the segregation patterns and markers GMI_ES17_c6425_188; GMI_ES15_c7632_384 were suggested by our map as potential for marker assisted selection. We observed lower frequency of the resistant SNP in the spring sown sub-population of the Collaborative Oat Research Enterprise (CORE) for the markers GMI_ES17_c6425_188 (10%) and GMI_ES02_C37788_255 (13%), suggesting these markers will be polymorphic in many populations segregating for PI258731 resistance, making them good candidates for marker assisted selection. As mentioned above, the marker GMI_ES02_C37788_255 did not segregate in our *A*. *strigosa* population but could have the same utility in differentiating *A*. *strigosa* and *A*. *sativa* germplasm regardless of the *A*. *strigosa* segment introgressed into hexaploid oat. However, marker (GMI_ES15_c7632_384) may have limited utility for marker assisted selection. A breeder would need to examine the genotypes of their parental lines in their breeding program and may need to select an alternative linked marker or may need to develop an assay for an alternate linked marker.

Linked markers have the potential to diagnose whether other *A*. *strigosa* accessions carry the PI258731 resistance. A marker or marker haplotype would be diagnostic if it accurately differentiated the PI258731 resistance from that of all other types of resistance detectable using crown rust races. We were unable to observe a unique resistant pattern with the available isolates, and so have not been able to evaluate marker patterns of their diagnostic potential. To achieve the goal to identify diagnostic marker or marker haplotype, more isolates would be required and need to do comparative sequencing to identify additional markers tightly linked to, and in LD with the resistant allele [8].

## Conclusion

In summary, we mapped the crown rust resistance gene using oat iSelect 6K SNP chip that placed the PI258731 resistance to two putative loci (in response to two separate *Pca* races TTTG and QTRG) between 4.1 cM (GMI_DS_LB_3547) and 12.1 cM (GMI_ES05_c367_141; GMI_ES15_c7632_384) on LG 7 in a *A*. *strigosa*, bi-parental population inoculated with two *Pca* races TTTG and QTRG. It was evident that the seedling resistance was completely responsible for the field resistance that we observed in Baton Rouge, LA, Gainesville, FL and the Matt Moore buckthorn nursery, MN. Our data proposed SNP marker (GMI_ES17_c6425_188) a candidate for use in MAS, in addition to the marker suggested by Rines et al. (GMI_ES02_C37788_255). These markers will be valuable in breeding efforts that involve with introgressing the *A*. *strigosa* PI258731 source of crown rust resistance.

## Supporting information

S1 File (XLSX)
